# Determination of Dynamic Elastic Properties of 3D-Printed Nylon 12CF Using Impulse Excitation of Vibration

**DOI:** 10.3390/polym17152135

**Published:** 2025-08-04

**Authors:** Pedro F. Garcia, Armando Ramalho, Joel C. Vasco, Rui B. Ruben, Carlos Capela

**Affiliations:** 1 ESTG-IPLeiria—School of Technology and Management, Polytechnic Institute of Leiria, 2411-901 Leiria, Portugal; joel.vasco@ipleiria.pt (J.C.V.); rui.ruben@ipleiria.pt (R.B.R.); carlos.capela@ipleiria.pt (C.C.); 2 Polytechnic University of Castelo Branco, Av. Pedro Álvares Cabral, nº 12, 6000-084 Castelo Branco, Portugal; aramalho@ipcb.pt; 3 Centre for Mechanical Engineering, Materials and Processes (CEMMPRE), ARISE, Department of Me-chanical Engineering, University of Coimbra, Pólo II, Rua Luís Reis Santos, 3030-788 Coimbra, Portugal; 4 CDRSP—Center for Rapid and Sustainable Product Development, Polytechnic Institute of Leiria, 2430-028 Marinha Grande, Portugal

**Keywords:** impulse excitation of vibration (IEV), nylon 12CF, fused deposition modeling (FDM), anisotropic mechanical properties

## Abstract

Material Extrusion (MEX) process is increasingly used to fabricate components for structural applications, driven by the availability of advanced materials and greater industrial adoption. In these contexts, understanding the mechanical performance of printed parts is crucial. However, conventional methods for assessing anisotropic elastic behavior often rely on expensive equipment and time-consuming procedures. The aim of this study is to evaluate the applicability of the impulse excitation of vibration (IEV) in characterizing the dynamic mechanical properties of a 3D-printed composite material. Tensile tests were also performed to compare quasi-static properties with the dynamic ones obtained through IEV. The tested material, Nylon 12CF, contains 35% short carbon fibers by weight and is commercially available from Stratasys. It is used in the fused deposition modeling (FDM) process, a Material Extrusion technology, and exhibits anisotropic mechanical properties. This is further reinforced by the filament deposition process, which affects the mechanical response of printed parts. Young’s modulus obtained in the direction perpendicular to the deposition plane (E_33_), obtained via IEV, was 14.77% higher than the value in the technical datasheet. Comparing methods, the Young’s modulus obtained in the deposition plane, in an inclined direction of 45 degrees in relation to the deposition direction (E_45_), showed a 22.95% difference between IEV and tensile tests, while Poisson’s ratio in the deposition plane (v_12_) differed by 6.78%. This data is critical for designing parts subject to demanding service conditions, and the results obtained (orthotropic elastic properties) can be used in finite element simulation software. Ultimately, this work reinforces the potential of the IEV method as an accessible and consistent alternative for characterizing the anisotropic properties of components produced through additive manufacturing (AM).

## 1. Introduction

Additive manufacturing (AM) has had a significant evolution, both in domestic and industrial use. It can be defined as a process that allows, based on a digital 3D model (obtained by CAD or reverse engineering), the construction of physical objects with complex geometry through the layer-by-layer deposition of material(s). Currently, AM is capable of building engineering parts (with applied requests), medical implants, and even artificial organs [1]. It is a manufacturing method especially suitable when single/customizable models and/or small series are demanded [2]. In these processes, a variety of materials with large differences in mechanical properties can be used [1]. Since it is a process with the ability to apply the amount of material appropriate to the request (geometry resulting from topological optimization) and because it generates very low waste, AM processes are seen as technologies with a high degree of sustainability [3,4].

Predicting the mechanical strength of parts built by AM is a challenging task, due to the complex geometries (some of which can only be obtained through this type of technology) and, essentially, due to the diverse methods of materialization of the component, the construction layer-by-layer, generating anisotropy in the component [5]. Anisotropy can be increased if the filament itself is also anisotropic, in the case of containing fibers dispersed in the thermoplastic matrix [6].

In most studies, the characterization of materials obtained via MEX relies on classic tensile and shear tests, specifically ASTM D638-14 [7], ASTM D5379/D5379M-19 [8] and D3518/D3518M-18 [9]. To obtain the PLA properties obtained by MEX, Song et al. [10] assume that the material is considered orthotropic in the plane of deposition, while with respect to the build direction, the properties are obtained by assuming the material as transversely isotropic. However, relying solely on classical tests for the mechanical characterization of anisotropic materials may not always be ideal due to economic constraints, time requirements, accuracy demands, and technological challenges [11]. While static tensile tests remain the gold standard for determining Young’s modulus, their destructive nature and resource demands highlight the need for non-destructive alternatives such as the IEV, which enables fast and reliable characterization using minimal equipment [12].

ASTM E1876-22 proposes a method, non-destructive testing (NDT), to determine the dynamic elastic properties of materials using vibration impulse excitation at room temperature. Although impulse excitation vibration has been applied to characterize the elastic properties of isotropic materials [13], it is worth mentioning that its application to anisotropic materials is rare [14,15]. One study was found where vibration impulse excitation was applied to the polylactic acid obtained by the MEX process [11]. More recently, this technique has been applied to the characterization of the elastic properties of a laminated composite [16].

The design of products with complex geometries increasingly relies on the development of numerical models using the finite element method (FEM), with it being necessary, for this purpose, to characterize the constitutive model of the material [17]. These models allow for simulating the mechanical behavior of components during service. While there are numerous studies that employ the FEM to characterize the mechanical behavior of 3D-printed components, most of these assume isotropic materials [18,19]. In contrast, studies that incorporate anisotropic material behavior into numerical finite element models are less common and typically focus on simpler geometries [20,21]. More recently, failure criteria traditionally used in laminated composites have been successfully applied to failure prediction of more complex geometries obtained by MEX [17].

Although IEV has been widely applied to mechanical characterization of metals [22], composites [23], and ceramics [24], its use in the characterization of anisotropic 3D-printed polymers remains limited, especially in fiber-reinforced thermoplastics, where directional stiffness plays a critical role. This study represents, to the best of our knowledge, the first application and validation of IEV in the characterization of a short-fiber-reinforced polymer manufactured via 3D printing.

In this article, a study is presented in which the anisotropic dynamic elastic properties of Nylon 12CF components materialized by MEX are obtained through the impulse excitation vibration. The obtained results are validated through classic tensile tests, ensuring their reliability and accuracy.

## 2. Background and Theory on IEV

**Figure 1:**
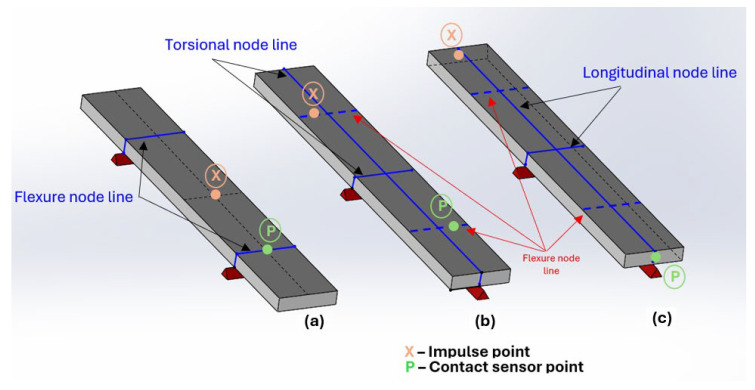
Boundary conditions imposed on the rectangular specimen for the excitation of the fundamental vibration mode: (**a**) flexional, (**b**) torsional, and (**c**) longitudinal.

According to ASTM E1876-22 [25], the technique allows the determination of the modulus of elasticity of the material based on the natural frequency of a specimen with a geometry in the shape of a bar, cylinder, disc, or ring. The natural frequencies are excited through a small mechanical impulse (impact), and then the response data will be acquired through an acoustic or piezoelectric sensor. The signals obtained will undergo a mathematical processing of the signal to obtain the spectrum of resonant frequencies. The determinations of the dynamic elastic moduli (and consequent calculation of the Poisson’s ratio) will be calculated using the equations provided by the ASTM standard, which considers the frequencies obtained (recorded by the software), the mass of the specimen, the type of specimen geometry, and its dimensions [26]. The ideal boundary conditions for the main vibration modes in rectangular specimens are illustrated in Figure 1, which indicates the positions of the supports, the impact location, and the resonance response reading [27].

In the measurement of the flexional resonance frequency, the specimen should be supported on supports that are distanced 0.224 L from each end (where L corresponds to the total length of the specimen), longitudinally. In the case of torsional frequencies, the specimen should be supported on the nodal lines at 0.5 L, both longitudinally and transversely. This will be the line that divides the length of the specimen equally, and there will be another that will divide it by its width. Finally, at longitudinal frequencies, the specimen must be supported in the same way as described for the torsional frequency mode, with the only change in the test being the position of impact and the resonance frequency reading [25,28].

In flexional vibration mode, the determination of the dynamic modulus of elasticity, in Pa, is defined by Equation (1):

(1) $$E\;=\;0.9465\times \left(\frac{m\times f_{f}^{2}}{b}\right)\times \left(\frac{L^{3}}{t^{3}}\right)\times T_{1}$$

where $f_{f}$ is the resonant frequency of the first bending mode (Hz), m is the mass of the specimen (g), b its width (mm), L its length (mm) t its thickness (mm), and $T_{1}$ a correction factor (dimensionless). As the ratio $\frac{L}{t}$ will be greater than 20, for the specimens that were manufactured for this test, the correction factor $T_{1}$ will be calculated using Equation (2):

(2) $$T_{1}\;=\;\left[1.000+6.585\times {\left(\frac{t}{L}\right)}^{2}\right]$$

In this way, it will not be necessary to know the Poisson ratio beforehand, which, in case the ratio $\frac{L}{t}$ is less than 20, would be imperative for its calculation.

In torsional vibration mode, the shear modulus, in Pa, can be calculated using Equation (3):

(3) $$G\;=\;\frac{4\times L\times m\times f_{t}^{2}}{b\times t}\times \left[\frac{B}{\left(1+A\right)}\right]$$

where $f_{t}$ is the fundamental resonant frequency of the specimen in torsion (Hz), and *B* (dimensionless) is a term that depends on geometry, defined by Equation (4):

(4) $$B\;=\;\left[\frac{\frac{b}{t}+\frac{t}{b}}{4\times \left(\frac{t}{b}\right)-2.52\times {\left(\frac{t}{b}\right)}^{2}+0.21\times {\left(\frac{t}{b}\right)}^{6}}\right]$$

*A* (dimensionless) is an empirical correction factor that depends on the ratio of width to thickness of the specimen and can be omitted if errors greater than 2% are admitted. It will then be calculated by Equation (5):

(5) $$A\;=\;\frac{\left[0.5062-0.8776\times \left(\frac{b}{t}\right)+0.3504\times {\left(\frac{b}{t}\right)}^{2}-0.0078\times {\left(\frac{b}{t}\right)}^{3}\right]}{\left[12.03\times \left(\frac{b}{t}\right)+9.892\times {\left(\frac{b}{t}\right)}^{2}\right]}$$

In longitudinal vibration mode, the modulus of elasticity obtained will refer to the orientation of the length of the specimen. Therefore, the orientation of the filaments in the specimen will determine which modulus is being determined ($E_{11},\;E_{22}$, or $E_{33}$). The dynamic modulus of elasticity, in Pa, can be calculated by Equation (6):

(6) $$E\;=\;\;4\times m\;\times f_{l}^{2}\times \left[\frac{L}{b\times t\times K}\right]$$

in which $f_{l}$ is the fundamental longitudinal frequency of the specimen (Hz). K (dimensionless) is a correction factor that depends on the Poisson’s ratio, v, and dimensions of the specimen, determined by Equation (7):

(7) $$k\;=\;1-\left[\frac{\pi^{2}\times v^{2}\times D}{8\times L^{2}}\right]$$

in which D (mm^2^) corresponds to a correction factor that depends on the width and thickness of the specimen, determined by Equation (8):

(8) $$D\;=\;\frac{2}{3}\times \left(b^{2}+t^{2}\right)$$

The characterization of Poisson’s ratio via the impulse excitation vibration is performed indirectly. It is obtained either through the correlation between Young’s moduli and shear moduli or by applying the reciprocity equation for Poisson’s ratios. These equations are derived from the Theory of Elasticity and are directly related to the stiffness matrix, which reflects the symmetry inherent in the specimen [26]. The relevant equations, presented as Equations (9)–(11), are shown below:

- Isotropic material:

(9) $$v=\frac{E}{2\times G}-1$$

- Material transversely isotropic:

(10) $$v_{23}=\frac{E_{22}}{2\times G_{23}}-1$$

- Orthotropic material:

(11) $$\frac{v_{12}}{E_{11}}=\frac{v_{21}}{E_{22}}\;\;\;,\;\;\;\frac{v_{13}}{E_{11}}=\frac{v_{31}}{E_{33}}\;\;\;,\;\;\;\frac{v_{23}}{E_{22}}=\frac{v_{32}}{E_{33}}$$

## 3. Materials and Methods

The experimental tests were conducted in the laboratories of the Department of Mechanical Engineering at the Polytechnic Institute of Leiria, in the laboratories of Engineering and Industrial Management at the Polytechnic University of Castelo Branco, and in the laboratories of the Department of Mechanical Engineering at the University of Coimbra. This section provides information on the tested material, sample preparation, and test procedures.

### 3.1. Material

The tested specimens are made of Nylon 12CF, a registered trademark material by Stratasys (Rehovot, Israel). It is a filament-shaped material used in their additive manufacturing machines in the FDM process, specifically in the models Fortus 450mc and F900. This thermoplastic material is reinforced with short carbon fibers, consisting of 35% by weight of fibers with a length of 150 μm and a diameter of 8 μm [29]. Based on visual inspection of a scanning electron microscope (SEM) image from [29], obtained using a ZEISS (Oberkochen, Germany) SEM, a preferential alignment of fibers within the thermoplastic matrix along the extrusion direction can be observed.

**Figure 2:**
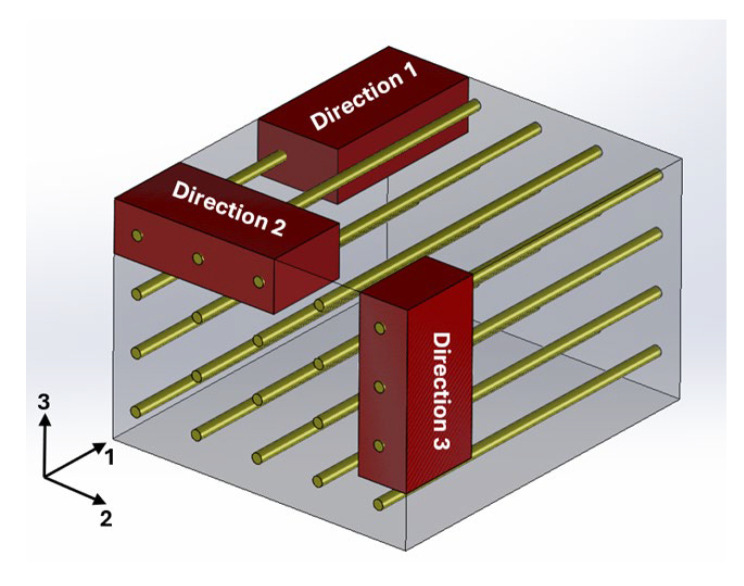
Generic representation of a structure detailing how the specimens should be obtained in the three main directions for non-isotropic materials.

Composite materials with continuous and oriented filaments are typically classified as orthotropic, displaying distinct mechanical properties depending on the direction of analysis. To apply the IEV and perform tensile tests on such materials, it is essential to prepare specimens with varied orientations of the composite structure, as illustrated in Figure 2. For instance, one can consider the fibers or filaments to be aligned along direction 1 of the reference depicted in Figure 2.

The specimens were manufactured using a Stratasys Fortus 900 machine (Stratasys, Rehovot, Israel), which has a build area of 610 mm × 914 mm and a height of 914 mm. The corresponding machine parameters used in the fabrication process are summarized in Table 1.

The production of specimens using this machine and material requires a thin sheet of transparent nylon (a Stratasys material), which is fixed to the build platform by vacuum. The parts are fabricated directly on this sheet. The machine has a particular feature when working with Nylon 12CF: even if the part does not structurally require supports, they are automatically generated to facilitate part removal from the nylon sheet.

In the post-processing step, the support material is removed by dissolution in an aqueous solution with the Stratasys chemical agent P400SC. The pieces are submerged in this bath in a tank heated up to 50 °C for 4 h.

**Figure 3:**
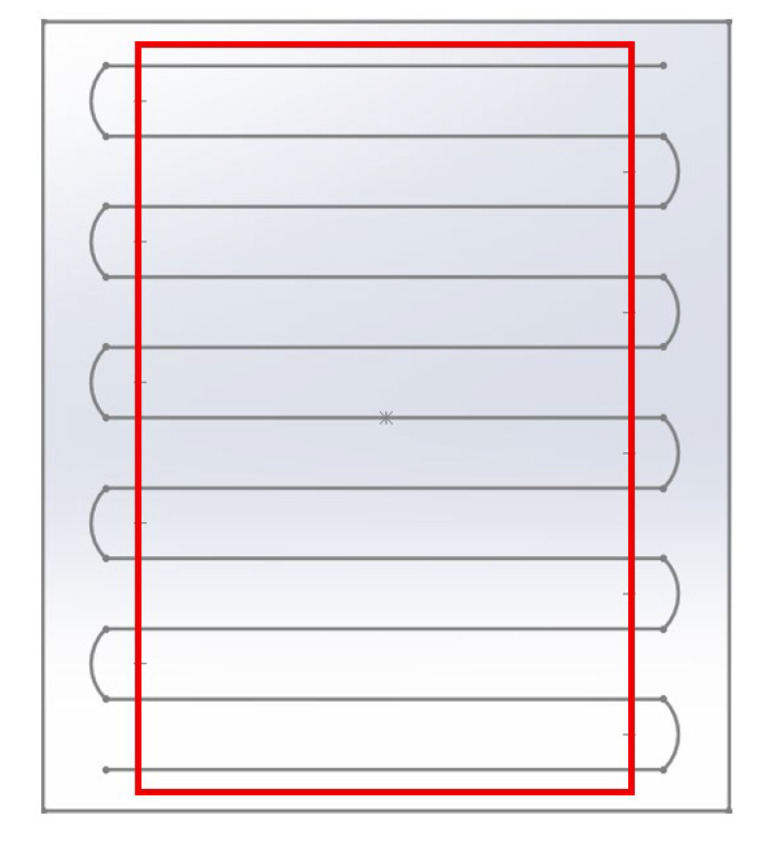
Representation of a single layer of the perimeter filament and the transition of direction of the filament deposition in the FDM process.

As mentioned in Chapter 2, the specimens must strictly adhere to the ratio of $\frac{L}{t}$ being ≥20. Therefore, the specimens were produced with the initial dimensions of 7 × 38 × 138 (mm). In the FDM process, for a part in production, there will be one or several filaments that are deposited to form the perimeter of the part, and there is also a “zig zag” zone in the transition of the filament deposition direction, as represented in Figure 3.

**Figure 4:**
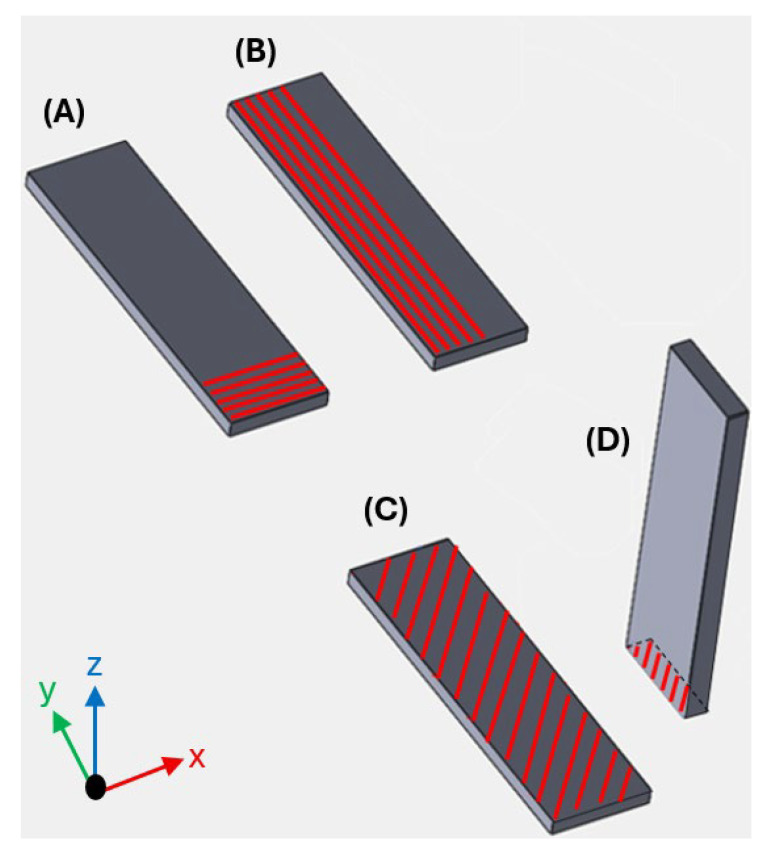
Specimens for IEV (base of construction in the X-Y plane): (**A**) deposition of the filaments at 0°, perpendicular to the length of the specimen; (**B**) deposition of the filaments oriented at 90°, parallel to the length of the specimen; (**C**) deposition of the filaments at ±45°, flat; (**D**) deposition of the filaments at ±45°, upright.

For the analysis to focus solely on filaments aligned according to the desired directions, it is necessary to extract the interior geometry within the red marker, as represented in Figure 3. This ensures a higher-quality test, as the modulus of elasticity and Poisson’s ratio are accurately determined under conditions where all filaments are fully aligned in the same reference direction. Accordingly, the specimens were initially produced with construction and deposition directions, as illustrated in Figure 4, but were later machined to eliminate geometric effects.

The X-Y plane represents the construction base of the machine, and each red line represents a filament, where successive ones will form a layer. In specimen A, all layers have the deposition of the filaments at 0°, perpendicular to the length of the specimen. In specimen B, the layers have the filaments oriented at 90°, parallel to the length of the specimen. For specimens C and D, the layers will be at ±45°. It should be noted that all the specimens in this study were manufactured with 100% infill density.

**Figure 5:**
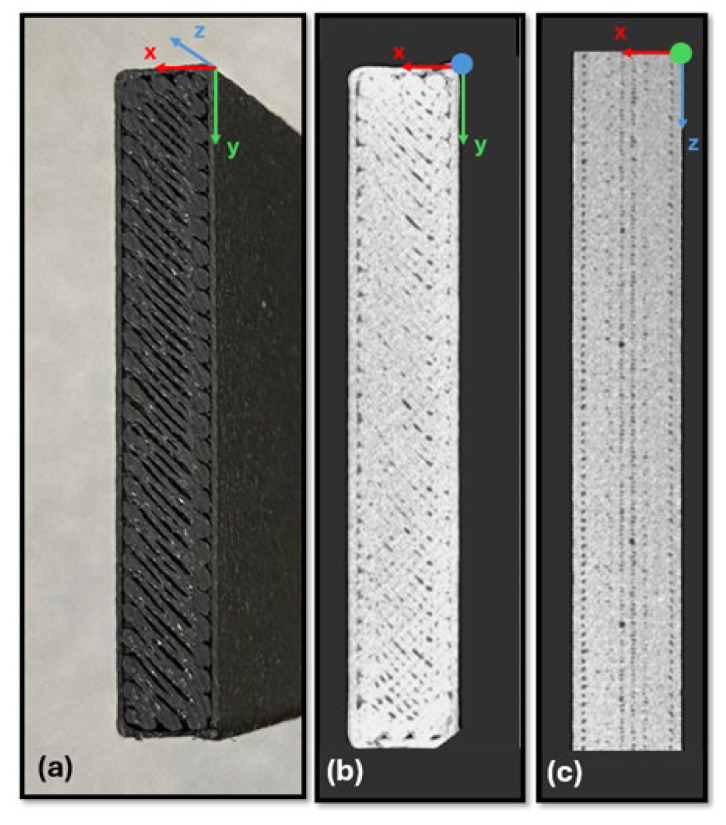
(**a**) Photo of specimen D. (**b**) Micro-CT of specimen D in the X-Y plane. (**c**) Micro-CT of specimen D in the X-Z plane.

At the end of the process of materialization of the specimens, it was noted that the layers that formed the perimeter did not have a complete aggregation to the core of the pieces produced. To this end, a Micro-CT analysis was performed to better understand the effect that occurred. The Phoenix v|tomex|x m 240 Micro-CT equipment (Baker Hughes, Houston, TX, USA) was used, equipped with a flat panel detector with a resolution of 2024 × 2024 pixels, and the voltage and current of the X-ray emitter (conical configuration) were 100 kV and 400 μA, respectively. A total of 1194 projections were made in each analysis, the exposure time was adjusted to 131 ms, and the voxel size (spatial resolution) was 47 μm. Subsequently, grayscale projections were processed to reconstruct complete 3D images of all specimens (the volume) using the Phoenix Data X2 Reconstruction software version 2.6.0. Figure 5b,c shows the visual result of the 3D reconstruction of specimen D (Figure 5a) using the myVGL viewer version 2024.4.

The existing voids, the effect of transition of the direction of deposition, and the perimeter of the specimen are perceptible in the figures from the Micro-CT analysis. Figure 5c highlights the existence of pores that recur along the vertical sides of specimen D, between the deposited perimeter and the core of the specimen. The application of the IEV test on these layers that do not present great material continuity would impair the test. Thus, the importance of removing transition effects from the direction of deposition of the filaments and the perimeter is reinforced.

For the tensile test, the specimens were obtained by machining a plate of the material under study, measuring 177 mm in length, 131 mm in width, and 4 mm in thickness. The layers were deposited with alternating orientations of −45° and +45°, a pattern that was repeated throughout the construction. It should be noted that the plate produced was also manufactured with a 100% infill density, and the same machine parameters were used in the production of IEV specimens. Machining, in addition to generating the specimen’ geometry according to the chosen tensile test standard, was used to eliminate the previously mentioned undesirable effects.

### 3.2. Impulse Excitation of Vibration Testing

#### 3.2.1. Testing Equipment

For the IEV test, it is necessary to use a data acquisition system capable of recording the vibrations of the sample to obtain the resonant frequency. The vibration movement in time was collected through a thin piezoelectric disc contact transductor, which works using the bimorph effect. The generated electrical signal (voltage) was collected in the PicoScope 3204A (Pico Technology, St Neots, UK), connected to a laptop to acquire this information in the equipment’s software.

#### 3.2.2. Specimen Preparation

It is easily understood that the accuracy in the calculation of the dynamic elastic modulus in Equation (1) is highly dependent on the regularity of the specimen and on the uncertainty related to the dimensions (measurement). If it is observed that the variables of thickness (t) and specimen length (L) have an exponent of 3, then an error of 1% in the measurement of these dimensions will result in a 3% error in the estimated modulus.

Therefore, to reduce the variations of these dimensions in each specimen, the surfaces were polished to regularize them. The preparation of the specimens also involved marking the nodal lines along which each specimen must be supported during the test to isolate the different types of fundamental vibration modes to be tested (bending and twisting). The impact site marking and sensor position were also marked according to the ASTM E1876-22 recommendations presented in Section 2 and Figure 1.

#### 3.2.3. Testing Procedure

The attachment of the bimorph effect piezoelectric sensor to the specimen was performed by using a 2-component cold-curing resin. However, for each test carried out, it was necessary to use a new sensor, in addition to the possible damage imposed on the specimen after its removal. The method used to fix the sensor was through double-sided adhesive tape. In [30], the results obtained through tests using each of the fixation techniques presented above were compared. They found that the resonance frequency differed by less than 1.2% between the two forms of fixation for the various specimen materials they tested.

**Figure 6:**
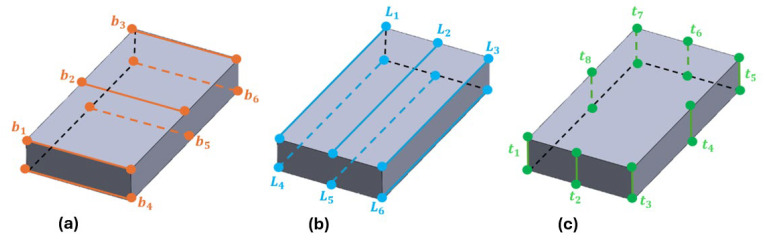
Location of the 20 zones to be measured as micrometer gauge for each specimen. There were (**a**) 6 measurements of the specimen width, (**b**) 6 measurements of the length, and (**c**) 8 measurements of the thickness of the specimen.

Before starting the test itself, the mass and dimensions of the specimen had to be measured precisely for the consequent calculation of the dynamic elastic properties. The mass of all specimens was determined using an electronic scale with an accuracy of 0.0005 g, meeting the requirement of 0.1% of the mass of the specimen stipulated in the ASTM E1876-22 [25]. Each dimension of the specimen was assumed based on the average of multiple readings along each of the sites shown in Figure 6.

The dimensions were acquired by micrometer gauge with a precision of 0.01 mm. The multiple measurements were not only used to calculate the average of each of the dimensions, but also to quantify the variation in dimensions throughout the specimen. The mean values of the specimens’ mass and geometric dimensions are presented in Table 2.

The tests were performed on a single sample of each specimen. However, since IEV is a non-destructive technique, three tests were performed on each specimen. This methodology has been followed by several authors in the application of IEV [16,31,32].

IEV testing simply involves applying a thrust (impact) to the specified location using an impact tool that meets the requirements stated in [25]. In practice, the size and geometry of the tool depends on the size and weight of the sample and the force required to produce vibration. For the dimensions of the specimens used, already presented in Table 2, a 5 mm diameter hard steel ball (balls used in bearings) was glued to a semi-rigid polymer bar. It must be ensured that the impact is not strong enough that the recorded amplitudes fall outside the measurable range of the sensor and/or software and that the impact does not create a bounce (double impact) on the specimen. To this end, a quick and light impact will be preferable to avoid such inconveniences.

For each type of IEV test, vibration signals were recorded by the PicoScope Software version 7 T&M. The configuration used in all IEV tests was 10,000 samples on a time basis of 50 ms and with the signal variation of −2 to 2 Volts. Using the Origin2017 software version Pro 2017 SR2, the fast Fourier transform was performed using the PicoScope values in text format (2 columns). Through this mathematical transformation, it is possible to move from the domain of time to the domain of frequency.

Through these 2 types of frequencies ($f_{f}$ and $f_{t}$) and using the equations described in Section 2, it is possible to obtain the longitudinal modulus of elasticity (E) and shear modulus (G), both dynamic moduli. For literature [33], through Equation (12), it is possible to determine the Poisson’s ratio of specimen C (constructed by layers ±45°) through the knowledge of the modulus of elasticity of specimens A ($E_{22}$) and B ($E_{11}$), from shear modulus ($G_{12}$), and from the longitudinal modulus of elasticity ($E_{45}$) obtained from specimen C.

(12) $$v_{12}=\frac{E_{1}}{2}\left(\frac{1}{G_{12}}-\frac{4}{E_{45}}+\frac{1}{E_{22}}+\frac{1}{E_{11}}\right)$$

This equation, initially defined for fiber-reinforced composite materials and in which its construction in the 1–2 plane was made using a symmetry (transversely isotropic piece in direction 3), can be used in the generic MEX process. Other authors have also used this equation to determine mechanical properties of parts produced by MEX in PLA material and assumed an elastic response of the transversely isotropic material [10]. To calculate the Poisson’s ratio, $\;v_{23}$, assuming isotropic transversality, Equation (10) was used.

### 3.3. Uniaxial Tensile Test

#### 3.3.1. Testing Equipment

For the mechanical tensile test, the Shimadzu AGS-100KNX universal testing machine (Shimadzu, Kyoto, Japan) was used, with a 100 KN load cell and equipped with wedge grips. To measure the transverse elongation in the tensile test of each tested specimen, a strain gauge of the type GFLA-3-350-50 from Tokyo Sokki Kenkyujo (Tokyo, Japan) was used. The Hottinger Baldwin Messtechnik (Darmstadt, Germany) extensometric bridge was used, which was responsible for making the entire electrical circuit of the Wheatstone bridge. The PicoScope 3204A (oscilloscope) (Pico Technology, St Neots, UK) was used to read the values of the extension variation and by recording it from the strain gauge. For the measurement of the longitudinal elongation, a clip-on extensometer was used, with the signal recording made through the testing machine’s own controller.

#### 3.3.2. Specimen Preparation

**Figure 7:**
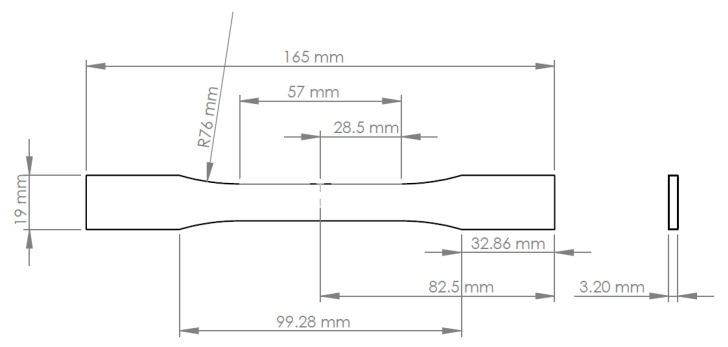
Type I specimen geometry was used for tensile testing, in accordance with ASTM D638-14.

The ASTM D638-14 standard was used for the mechanical tensile test. Five type I specimens were produced, meeting the dimensions specified in the standard, as shown in Figure 7.

After machining the plate of the material, using a Roland MDX-650 CNC (Roland DG Corporation, Hamamatsu, Japan) to obtain the geometry of the specimens, they were polished to ensure a good surface finishing for bonding the extensometers. Using the micrometer, the specimens were measured in the main dimensions for subsequent data processing after the tensile test. Several measurements were made to obtain the arithmetic mean of each dimension, as shown in Table 3.

Before starting the attachment of the strain gauge, its symmetry lines were marked on the specimen using a scriber. The surface of the specimen that would receive the strain gauge through petroleum ether was cleaned. With the help of a magnifying glass, the strain gauge was placed in the right position, depending on the markings that the strain gauge provided and the lines previously marked on the specimen. Cyanoacrylate adhesive was used for attaching the strain gauge to the surface of the specimen [34].

#### 3.3.3. Testing Procedure

**Figure 8:**
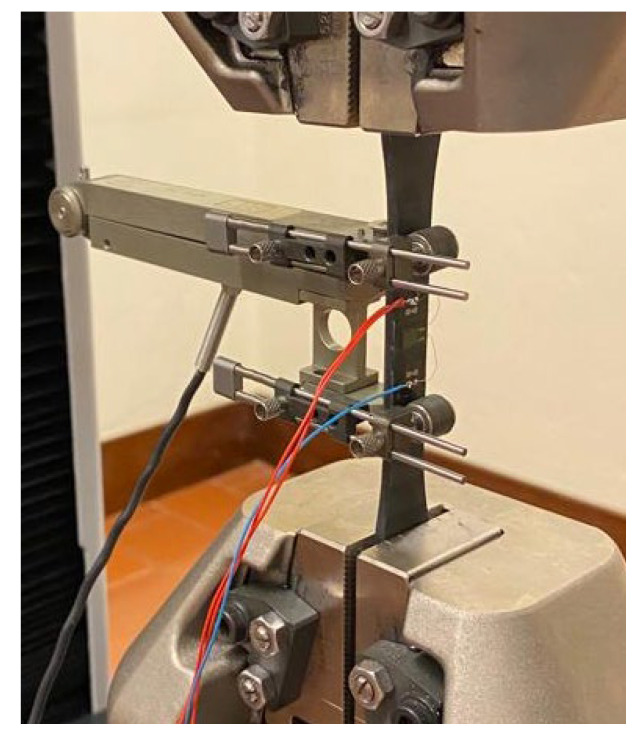
Clip-on extensometer and strain gauge glued to the specimen to be tested.

In the tensile test, according to ASTM D638-14, a test speed of 1 mm/min was applied to type I specimens, with an initial distance between grips of 115 mm. It was necessary to use the Wheatstone bridge to read the variation of the resistance in the strain gauge, and consequently, it was possible to determine the transverse elongation in the specimen. To measure the longitudinal elongation, the clip-on extensometer with an initial distance (gauge length) of 50 mm was used. Figure 8 shows the clip-on extensometer positioned on the specimen, the specimen with the strain gauge previously glued, and the various electrical wires that will be connected to measuring equipment.

The clip-on extensometer was connected directly to the controller of the tensile testing machine, which enabled us to obtain, at the end of the test, a data file containing the column of the duration of the test, the force measured by the load cell, the elongation of the specimen between grips, and also the elongation value recorded by the clip-on extensometer in the region of the gauge length of the specimen (value of higher accuracy).

The measurement and recording of strain gauge values followed a different approach. The strain gauge was connected to the Hottinger Baldwin Messtechnik strain gauge bridge (Germany), which managed the entire Wheatstone bridge electrical circuit, configured as a 1/4 bridge. From this device, one of its analog outputs was connected via cable to the PicoScope 3204A (Pico Technology, St Neots, UK), which was responsible for reading the variation in extension and recording the data. Simultaneously, another channel of the PicoScope was connected to the load cell of the tensile testing machine, capturing and recording the force values.

In this way, each of the two data files will contain a “constant” that allows for overlaying all the information. Thus, the same data column (force) is present in both files (force/transverse elongation and force/longitudinal elongation), enabling the calculation of the Poisson’s ratio according to the ASTM E132-04 standard [35].

## 4. Results

**Figure 9:**
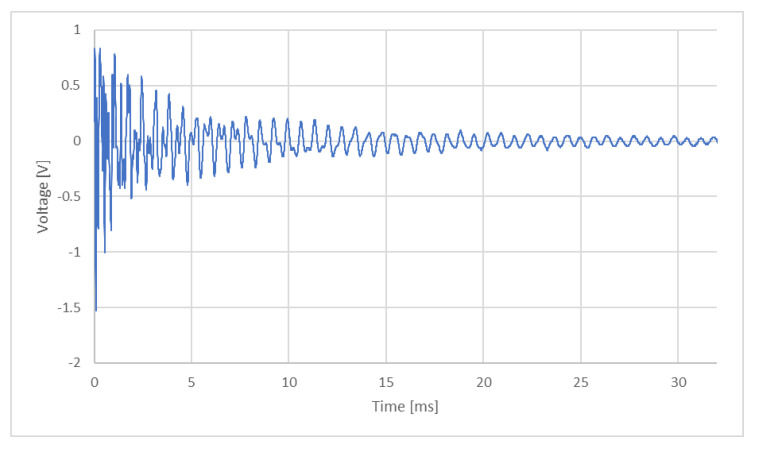
Voltage (V)—time (ms) history for the flexural vibration test mode on specimen A.

When testing specimen A in the flexural vibration test mode, the graph presented in Figure 9 was obtained, where time (in milliseconds) is represented on the *x*-axis and the voltage recorded by the piezoelectric sensor (in Volts) on the *y*-axis. This voltage reflects the propagation of vibration through the material from the impact zone to the sensor, allowing the dynamic characteristics of the sample to be assessed.

**Figure 10:**
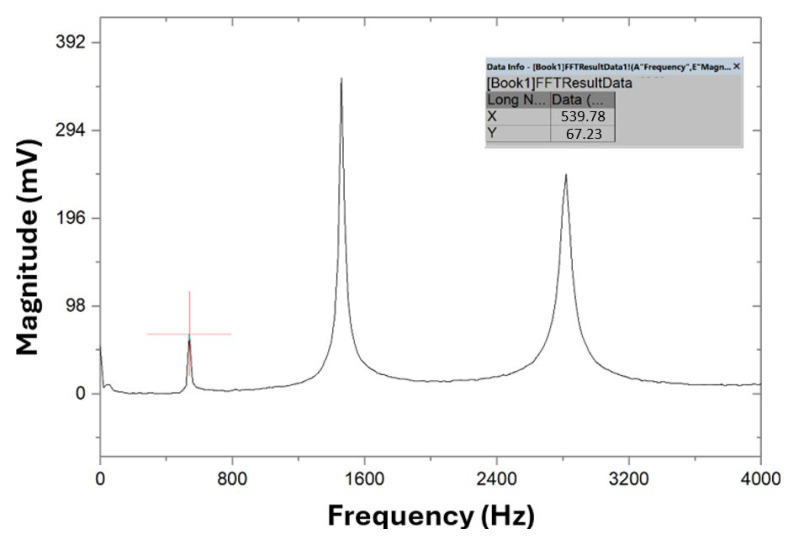
Frequency spectrum in the test of the flexural vibration mode on specimen A.

Using Origin2017 software, a fast Fourier transform (FFT) was applied to the data acquired via PicoScope, originally presented in a two-column text format. This mathematical operation converts the signal from the time domain to the frequency domain. The resulting frequency spectrum from the flexural vibration test is shown in Figure 10.

The main flexural resonance frequency ($f_{f}$) is determined from the first harmonic, corresponding to the first peak in the graph, with a value of 539.78 Hz. According to theory, from the integration of the differential equations of the vibratory motion of test specimens with parallelepiped geometry, with these boundary conditions, in isotropic and homogeneous materials, the frequencies of the second and third harmonics can be predicted by multiplying $f_{f}$ by the factors 2.7 and 5.4, respectively [36]. This prediction aids in identifying the main torsional frequency ($f_{t}$), as theoretically, the peak of the torsional vibration mode should be slightly to the left of the second harmonic of the flexural vibration test (1457.41 Hz).

**Figure 11:**
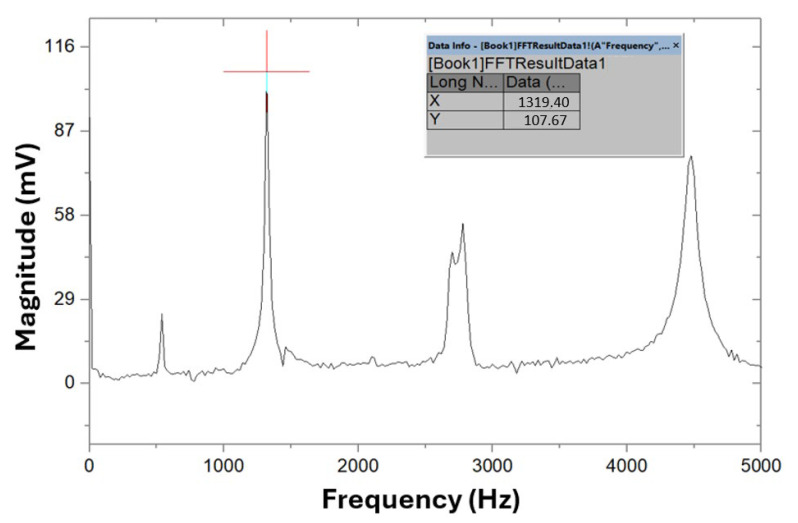
Frequency spectrum in the test of the torsional vibration mode on specimen A.

The process for determining the main torsional frequency ($f_{t}$) follows the same procedure, except for the positioning of the specimen on the supports, the location of the piezoelectric sensor, and the impact point. The closest peak to the left of the second harmonic of the flexural vibration test corresponds to a frequency of 1319.4 Hz, identified as $f_{t}$. Figure 11 presents the graph of the frequency spectrum obtained in the torsional vibration test.

Table 4 shows the frequency values obtained in the tests in flexional and torsional vibration modes for the specimens tested. There was great repeatability in the tests performed, and the frequencies obtained for the three tests performed for each of the test specimens were practically the same, so the test results were not distinguished, with only their average being presented.

Through these two types of frequencies and the equations described in Section 2, the mechanical properties (E, G) of the analyzed specimens can be obtained, as presented in Table 5, Table 6, Table 7 and Table 8, using the IEV.

Based on the flexural and torsional vibration tests of specimens A, B, C, and D, Table 9 shows the orthotropic properties of a piece built in the X-Y plane (1–2), with layers ±45°.

**Figure 12:**
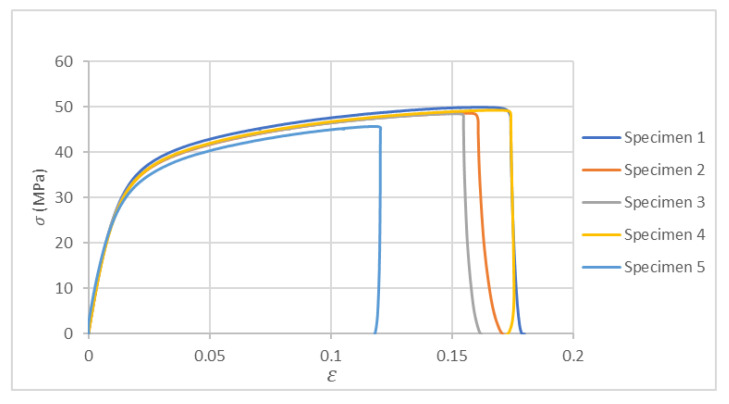
Stress–strain graph in the tensile test on Nylon 12CF.

Figure 12 presents the stress–strain graph obtained from the tensile test, illustrating the material’s mechanical response under applied load.

**Figure 13:**
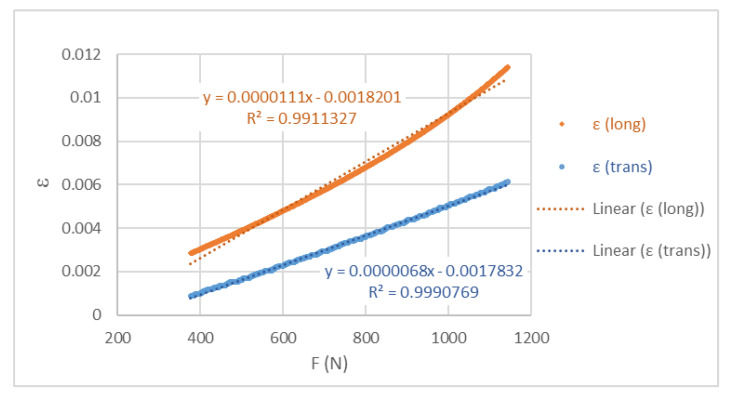
Graph of the longitudinal and transverse deformations of the traction specimen 1.

Figure 13 presents the graph of the longitudinal and transverse deformations of specimen 1 as an example. From the slopes of the graph lines, the Poisson’s ratio was determined, obtaining a value of 0.61.

Finally, Table 10 presents the key values extracted from each of the lines in the stress–strain graph, as well as the Poisson’s ratio corresponding to each of the tested specimens.

## 5. Discussion

The mechanical characterization of 3D-printed anisotropic materials requires careful consideration of both static and dynamic testing methods. In this study, tensile tests and impulse excitation of vibration (IEV) were used to evaluate key elastic properties.

To better understand the relationship between these techniques, Table 11 presents a direct comparison between the common data obtained from both methods.

The difference is justified by the testing speeds of each technique, as the static elastic modulus and the dynamic elastic modulus of a specimen are directly related to the material’s viscoelastic behavior. In other words, the material’s response varies according to the strain rate applied during the test. According to literature, the dynamic elastic modulus, determined by the IEV test, will always be equal to or greater than that obtained in the quasi-static tensile test [28,29]. This is because the strain rate in a dynamic test is always higher than that applied in static tests, while the strain levels imposed in a dynamic test are lower. In [37], the influence of the deformation rate on PLA obtained by the MEX process is analyzed using a universal material testing machine and a separated Hopkinson pressure bar experimental device, confirming the same trend. Furthermore, it is evident that strength properties can only be determined through standard tensile tests.

Compared to the data obtained in the tests performed and those provided by the Stratasys material data sheet [38], only one mechanical property can be compared. The Young’s modulus value, $E_{33}$ (3.52 GPa), obtained in the impulse excitation vibration, has the same meaning as the modulus of elasticity in the ZX specimen (3.00 GPa) of the technical sheet, where the difference in results is 14.77%.

In this study, three IEV application tests were performed on each of the specimens, which demonstrated constancy of resonance frequencies and, consequently, of the obtained dynamic elastic properties. Thus, although the variability of properties related to the IEV was considered, the same was not true for the variability of properties related to the sample. Based on the tensile test results, which yielded small standard deviations, we can conclude that sample variability would have little significance in this study. However, to evaluate the properties of the material obtained by MEX in situations where less homogeneity is expected, it is recommended to use a significant number of samples.

The impulse excitation of vibration, compared to tensile tests, is a way of characterizing the elastic properties of a material using equipment of lesser value. It is a much less recognized process; however, it has reliable values and high repeatability. Its application to anisotropic materials has essentially been to composite materials with continuous fibers and in a thermosetting matrix [16]. The application of this technique for the characterization of parts produced by additive manufacturing is of high academic and industrial interest. In the case of the MEX process, it is possible to estimate the elastic properties (E, G, v) in the three directions of the Cartesian reference frame of the material. The process is non-destructive and requires samples (specimens) of small dimensions. The process of obtaining data is of low simplicity, and the data transformation (using Fourier transform) can be performed using open software or not. The interpretation of the results, especially for anisotropic materials, requires some care and experience.

## 6. Conclusions

This study demonstrates the applicability of the impulse excitation of vibration for characterizing the anisotropic elastic properties of 3D-printed Nylon 12CF, produced via the MEX additive manufacturing process. The method allowed the estimation of key elastic properties in different material directions and was supported by comparative results from quasi-static tensile testing. The differences observed are consistent with the viscoelastic nature of the polymer matrix and the distinct strain rates of each technique.

Given its operational simplicity, low cost, and non-destructive nature, IEV presents itself as a promising alternative for mechanical characterization of anisotropic materials in both academic and industrial contexts. Its potential for broader application to other materials and processes still underexplored makes this contribution a relevant foundation for future studies and innovation in the design of functional components.

## Figures and Tables

**Table 1:** Machine parameters in the construction of specimens.

3D Printing Parameter	Value/Reference
Nozzle of Nylon 12CF material	T20C (Stratasys Reference)
Nozzle for support material (SR130)	T12 SR100 (Stratasys Reference)
Layer height	0.254 mm (only option in this material)
Nozzle temperature for Nylon 12CF	336 °C
Nozzle temperature for SR130	240 °C
Temperature of the build chamber	125 °C
Nozzle diameter for Nylon 12CF	(Info not available)
Nozzle diameter for SR130	(Info not available)

**Table 2:** Mean values of mass and geometric dimensions of the specimens tested.

Average Dimensions	Specimen A	Specimen B	Specimen C	Specimen D
L, length (mm)	133.56	116.83	133.20	137.07
b, width (mm)	33.75	32.72	35.13	33.92
t, thickness (mm)	6.16	3.01	3.49	4.40
m, mass (g)	31.4590	12.7721	18.3958	23.7136

**Table 3:** Measured dimensions of the tensile test specimens.

Dimensions (mm)	Specimen 1	Specimen 2	Specimen 3	Specimen 4	Specimen 5
$w_{1}$	12.98	13.01	12.97	12.99	12.96
$t_{1}$	3.21	3.23	3.12	3.22	2.99
$w_{2}$	12.98	13.01	12.98	12.99	12.98
$t_{2}$	3.23	3.24	3.08	3.21	3.01
$w_{3}$	12.98	13.01	12.96	12.98	12.98
$t_{3}$	3.24	3.24	3.12	3.23	3.02
Average width ($w_{avg}$)	12.98	13.01	12.97	12.99	12.97
Average thickness ($t_{avg}$)	3.23	3.24	3.11	3.22	3.01

**Table 4:** Frequency values obtained for the tests in flexional and torsional vibration modes of the specimens tested.

Type of Frequency	Specimen A	Specimen B	Specimen C	Specimen D
$f_{f}$ (Hz)	539.78	779.68	379.84	419.83
$f_{t}$ (Hz)	1319.4	1839.2	1099.5	899.64

**Table 5:** Mechanical properties obtained by the IEV in specimen A.

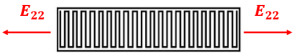	
Longitudinal modulus of elasticity, $E_{22}$	2.62 GPa
Shear modulus, G	1.04 GPa

**Table 6:** Mechanical properties obtained by the IEV in specimen B.

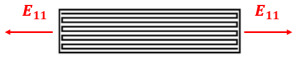	
Longitudinal modulus of elasticity, $E_{11}$	13.13 GPa
Shear modulus, G	6.37 GPa

**Table 7:** Mechanical properties obtained by the IEV in specimen C.

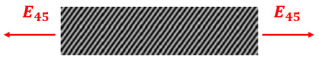	
Longitudinal modulus of elasticity, $E_{45}$	3.99 GPa
Shear modulus, $G_{12}$	2.60 GPa

**Table 8:** Mechanical properties obtained by the IEV in specimen D.

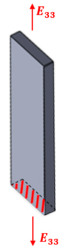	
Longitudinal modulus of elasticity, $E_{33}$	3.52 GPa
Shear modulus, $G_{23}$	1.14 GPa

**Table 9:** Orthotropic properties of a piece built in the X-Y plane (1–2) based on flexural and torsional vibration tests.

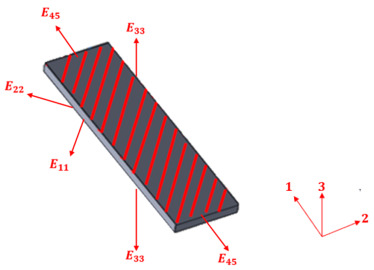	
Modulus of Elasticity, $E_{45}$ (MPa)	3987.27
Modulus of elasticity parallel to the direction of deposition of filaments, $E_{11}$ (MPa)	13,130.90
Modulus of elasticity perpendicular to the direction of filament deposition, $E_{22}$ (MPa)	2620.98
Modulus of elasticity in direction 3, $E_{33}$ (MPa)	3518.84
Shear modulus, $G_{12}$ (MPa)	2600.50
Shear modulus, $G_{23}$ (MPa)	1139.20
Poisson’s ratio, $v_{12}$	0.55
Poisson’s ratio, $v_{23}$	0.54

**Table 10:** Tensile test results of Nylon 12CF.

Mechanical Property	Specimen 1	Specimen 2	Specimen 3	Specimen 4	Specimen 5	Average	Standard Deviation
Young’s modulus, $E_{45}$ (MPa)	3068.6	3119.0	3102.2	3119.2	2945.6	3070.92	65.31
Yield strength (MPa)	31.8	30.6	30.9	30.4	29.9	30.71	0.63
Ultimate Strength (MPa)	49.9	48.7	48.5	49.3	45.7	48.42	1.45
Poisson’s ratio, $v_{12}$	0.61	0.58	0.61	0.62	0.53	0.59	0.0346

**Table 11:** Comparison between tensile test results and IEV data.

Mechanical Property	Tensile Test	IEV	Variation	Relative Variation
Young’s modulus, $E_{45}$	3070.92 MPa	3987.27 MPa	916.35 MPa	22.95%
Poisson’s ratio, $v_{12}$	0.59	0.55	0.04	6.78%

## Data Availability

The original contributions presented in this study are included in the article. Further inquiries can be directed to the corresponding author.
